# Glioma dataset from Rabat: Clinicopathological, immunohistochemical and disease progression features of 32 Moroccan patients with diffuse Glioma

**DOI:** 10.1016/j.dib.2022.108265

**Published:** 2022-05-12

**Authors:** Fatima Sfifou, Mounir Ouzir, El Mehdi Hakkou, Majdouline Obtel, Hassan Errihani, Abderrahmane Al Bouzidi, Redouane Abouqal, Abdessamad El Ouahabi, Nadia Cherradi

**Affiliations:** a Research's Pedagogic Unit of Pathological Anatomy, Laboratory of Pathological Anatomy, Research Team in Tumour Pathology, Faculty of Medicine and Pharmacy, Mohammed V University in Rabat, Avenue Mohammed Belarbi El Alaoui, Souissi, BP 6203, Rabat Institutes, Rabat 10000, Morocco; b Pathological Anatomy Department, Hospital of Specialities in Rabat, Morocco; c Department of Biology, Group of Research in Physiology and Physiopathology, Faculty of Sciences, Mohammed V University in Rabat, BP 1014 Rabat, Morocco; d Neurosurgery Department, Hospital of Specialities in Rabat, Morocco; e Laboratory of Biostatistics, Clinical Research and Epidemiology, Rabat Faculty of Medicine and Pharmacy, Mohammed V University in Rabat, Morocco; f National Oncology Centre Sidi Mohamed Ben Abdallah in Rabat, Morocco

**Keywords:** Glioma, Hypoxia, Immunohistochemistry, HIF-1alpha, IDH1, TP53, Disease progression

## Abstract

The Moroccan Glioma Dataset contains the clinical data of 32 patients with glioma. The clinical data including demographic data (age, sex), tumor characteristics (tumor location, Glioma type, Karnofsky performance score, mitotic activity, cell density, necrosis, endotheliocapillary vascular proliferation, MRI contrast pick-up, corpus collosum infiltration and Oedema), treatment strategy (subtotal resection, gross resection, biopsy, radiotherapy, chemotherapy), expression pattern of tumor biomarkers (IDH1, HIF-1alpha, P53, Ki-67), and survival data (Kaplan-Meier curves for disease progression). The dataset can be used to relate tumor characteristics to tumor biomarkers and to predict disease progression for a better treatment management. The data were presented, analyzed, and described in the article “Immunohistochemical expression of HIF-1α, IDH1 and TP53: prognostic profile of Moroccan patients with diffuse glioma” published in Journal of Chemical Neuroanatomy [1].

## Specifications Table

**Table:** 

Subject	Cancer research
Specific subject area	Prognostic biomarkers, hypoxia, WHO 2016 subtypes, patients with glioma, disease progression
Type of data	Text, table, figure
How the data were acquired	Patient follow up template was used to collect clinical data. Immunohistochemistry of HIF-1alpha, IDH1, TP53 and Ki-67 was carried out using formalin-fixed, paraffin-embedded tumors tissue obtained from biopsy or resection. Images of immunostaining were taken with a digital camera (Leica Application Suite Version 3.0) connected to optical microscope (LEICA DM750).
Data format	Raw and Analyzed
Description of data collection	Data were acquired following written consent from all the 32 glioma patients between June 2017 and December 2019. All patients underwent gross or subtotal resection, or biopsy. A survey was used for the initial collection of patient information. Preoperative imaging was acquired according to the routine clinical protocol. Immunostaining of IDH1, HIF-1alpha, p53, Ki-67, and grade of tumor were determined either as part of the treatment process or for research purposes.
Data source location	Institution: Pathological Anatomy and Neurosurgery Departments of the Rabat Specialities Hospital and the Sidi Mohamed Ben Abdallah National Oncology Centre in Rabat, Morocco City: Rabat Country: Morocco
Data accessibility	Repository name: Mendeley Data Data identification number: 10.17632/vkt6p2xztv.2 Direct link to the dataset: Glioma Dataset from Rabat: Clinicopathological, immunohistochemical and disease progression features of 32 Moroccan patients with Glioma. - Mendeley Data
Related research article	F. Sfifou, M. Ouzir, E.M. Hakkou, M.Obtel, H. Errihani, A. AL Bouzidi, R. Abouqal, A. El Ouahabi, N. Cherradi, Immunohistochemical expression of HIF-1α, IDH1 and TP53: prognostic profile of Moroccan patients with diffuse glioma. J Chem Neuroanat. 119 (2021), 102056, https://doi.org/10.1016/j.jchemneu.2021.102056

## Value of the Data

• This dataset provides clinical, radiological, histological, and immunohistochemical data, and outlined tumors from 32 patients with glioma. Currently, no data that provides all this information for a single Moroccan patient cohort is available.
• This data can be used to stratify patients according to their immunohistochemical profile, and to personalize treatment according to hypoxia marker expression, and to predict disease progression.
• These data may be used, or reused, in research that involves clinical features of diffuse glioma for comparative reasons, or to incorporate them as part of a larger dataset.

## 1 Data Description

The dataset consists of one Excel file, one word file (Patient follow up template) and Immunochemistry images. The Excel file contains the raw data and includes: patient ID, demographic data (age, sex), tumor characteristics (tumor location, glioma type, Karnofsky performance score, mitotic activity, cell density, necrosis, endotheliocapillary vascular proliferation, MRI contrast pick-up, corpus collosum infiltration and Oedema), treatment strategy (subtotal resection, gross resection, biopsy, radiotherapy, chemotherapy), expression pattern of tumor biomarkers (IDH1, HIF-1alpha, P53, Ki-67), and disease progression.

## 2 Experimental Design, Materials and Methods

### 2.1 Patients

A total of 71 consecutive patients with a potential diffuse glioma diagnosis on neuroimaging were considered for recruitment between June 2017 and December 2018. After pathologic examination, a total of 32 patients (22 with glioblastoma, and 10 with glioma grade II and III) were included in this study. All the cases underwent either surgical gross or subtotal resection or biopsy. The anatomopathological examination was carried out at the Pathological Anatomy Laboratory of the Specialities Hospital in Rabat, by experienced neuropathologists. Adjuvant chemotherapy and radiotherapy were fully discussed with patients before the start of treatment, and all patients had received adequate chemotherapy and radiotherapy according to the Stupp standard protocol for gliomas [2].

**Inclusion criteria**

• Male and female patients with histologically confirmed diffuse glioma WHO 2016.
• Patients of Moroccan nationality.
• Patients from the Hospital of Specialties, and the National Institute of Oncology in Rabat.
• Adult patients over the age of 18.
• Consenting patients.

**Exclusion Criteria**

• Patients with other brain tumors or brain metastasis.
• Patients over the age of 80.
• Patients of other nationalities.
• Patients with grade I glioma WHO 2016.

### 2.2 Histological Evaluation

The histological type and grade of the tumour were examined by a neuropathologist and determined according to the 2016 WHO Classification of Central Nervous System tumours [3]. The tumours tissue sections are stained with H&E (Hemalun Eosin) stains.

### 2.3 Immunohistochemistry for Paraffin Sections

After a dehydration step by immersion in alcohol baths of increasing concentrations (70, 80, 90 and then 100%), all tumor samples from biopsies or surgical specimens were fixed in 10% neutral buffered formalin for 2 to 24 h in room temperature, and after standard anatomopathological examination are embedded in paraffin.

*The other steps of the IHC is as follows*

- Paraffin sections (4-μm-thick) were cut by microtome and floated in water bath.
- The floating paraffin sections were mounted on chromo-gelatinized glass slides using a brush.
- Drying in the oven at 56 °C during 1 h
- Dewaxing in 2 baths of Toluene for 10 min each.
- Hydration with decreased concentration of alcohol (100%, 90%, 80%, and then 70%) for 5 min each, and tap water for 5 min.
- Antigen retrieval by immersion of the slides in sodium citrate buffer solution (at corresponding antibody pH) and heating in a microwave at 1200W for 5 min.
- The buffer and slides must cool down to room temperature before three times washing step in PBS (at corresponding antibody pH) for 5 min each.
- Demarcation of the sections by the PAP pen in order to delimit the field of the reactions
- Blocking of endogenous peroxidase activity by 3% hydrogen peroxide for 5 min followed by brief rinse in PBS.
- Incubation of the sections with primary antibody (anti-IDH1, anti-HIF-1-Alpha, anti-P53, or anti-Ki-67) in a humid box for 30 min at room temperature (anti-IDH1 was incubated overnight at a temperature of 4 °C).
- Incubation in the 2nd a biotin-conjugated (anti-mouse or anti-rabbit) antibody for 30–60 min and wash with PBS three times, 5 min for each wash.
- Incubation with a peroxidase-conjugated antibody and wash with PBS three times, 5 min for each wash.
- Addition of 1–5 drops of 3,3′ Diaminobenzidine (DAB) chromogen solution to cover the entire tissue section and incubate for 3–20 min until a brown color develops.
- -After rinsing the slides with PBS buffer, tissue sections were staining with Haris hemalin for 1 to 2 min.
- Slides are placed vertically on filter paper or a towel to allow them to dry.
- Visualization of staining of tissue under an optical microscope (LEICA DM750) and the images are taken with a digital camera (Leica Application Suite Version 3.0).

### 2.4 Reagents

- Mouse monoclonal antibody anti-HIF-1α (clone ESEE122, reference GR46837-1, 1:1000 dilution, pH 6) from Abcam;
- Mouse monoclonal anti IDH1R132H (clone H09, 1:40 dilution, pH 6) from Dia Nova;
- Mouse monoclonal anti p53 (clone DO-7, pre-diluted ready to use, pH 6) from Dako;
- Rabbit monoclonal anti Ki-67 (Ki-67 RMaB, clone: EP5, 7.0 ml, pre-diluted, ready-to-use, pH 7) from Bio SB.
- Citrate buffer (prepared extemporaneously)
- Tampon PBS (Phosphate Bufferd Saline)

### 2.5 Immunostaining Quantification

The expression of HIF-1*α* is assessed by counting the percentage of positive cells over the total cells in each slide. The staining of HIF-1α is evaluated as follows: staining is considered negative/weak staining when ≤10% of cells are positives and positive/strong staining of HIF-1α when >10% of cells are positives.

The Ki-67 index was estimated as the percentage of stained cell nuclei among at least 2000 tumor cells in the areas richest in positive cells (number of positive nuclei per 2000 cells). Tumors that express Ki-67 at levels of 2% or above were regarded as positive.

IDH1 immunoreactivity was considered “positive” when a large proportion of tumor cells showed high cytoplasmic reactivity, whereas it was considered “negative” if little or no cytoplasmic reactivity was observed.

The evaluation of p53 expression was performed semi-quantitatively by continuously counting more than 1000 tumor cells in the areas of highest expression. According to the percentage of tumor cells with reactivity, we considered p53 positive if the percentage of cells with positive reactivity over 10%, or low or negative reactivity if the percentage of cells is <10%.

### 2.6 Evaluation of Mitotic Activity

The estimation of the mitotic activity index was done on glass slides using Leica light microscopy where mitosis is counted in 10 high power fields (40× magnification). For a mitotic index of 6 mitoses or more per 10 high power fields, mitotic activity was considered present. The differentiation of true mitoses from apoptotic bodies, dark nuclei and tissue artefacts, requires careful work.

### 2.7 Disease Progression

The percentage of patients with disease progression was calculated from the time of surgery to one year, using one of the following criteria: an increase in tumor size of more than 20% in MRI and CT scan, the tumor has spread to healthy tissue, or the patient's condition worsens through paralysis or blindness. All patients were followed up every 3 months.

## Ethics Statements

The protocol was approved by the Ethics Committee of the Faculty of Medicine and Pharmacy, Mohammed V University in Rabat, Morocco (File N° 27/16) in accordance with the Helsinki Declaration (2008 Version), the International Ethical Guidelines for Biomedical Research Involving Human Subjects of the Council for International Organizations of Medical Sciences (CIOMS, 2002 Version), and the National Law (No. 28-13; 2015) for the Protection of Persons Participating in Biomedical Research. Informed consent was obtained from each of the participants.

## CRediT authorship contribution statement

**Fatima Sfifou:** Methodology, Data curation, Writing – original draft, Writing – review & editing. **Mounir Ouzir:** Formal analysis, Writing – original draft, Writing – review & editing. **El Mehdi Hakkou:** Data curation. **Majdouline Obtel:** Formal analysis. **Hassan Errihani:** Validation, Data curation. **Abderrahmane Al Bouzidi:** Conceptualization. **Redouane Abouqal:** Formal analysis, Validation. **Abdessamad El Ouahabi:** Validation, Data curation. **Nadia Cherradi:** Formal analysis, Validation, Writing – original draft.

## Declaration of Competing Interest

The author(s) declared no potential conflicts of interest with respect to the research, authorship, and/or publication of this article.

## Data Availability

Glioma Dataset from Rabat: Clinicopathological, immunohistochemical and disease progression features of 32 Moroccan patients with Glioma. (Original data) (Mendeley Data).

## Appendix Supplementary materials

Image, application 1
