# Supplementary material for: Glioma dataset from Rabat: Clinicopathological, immunohistochemical and disease progression features of 32 Moroccan patients with diffuse Glioma
Source: Data Brief. 2022 May 12;42:108265. doi: 10.1016/j.dib.2022.108265 (PMC9126762; doi:10.1016/j.dib.2022.108265)
Supplement: Supplementary file 1 [file mmc1.docx]

**
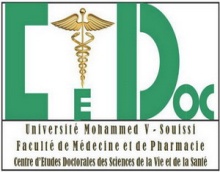
** **
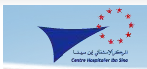
**

**Patient follow up template**

Hospital of specialties of Rabat

Department of Pathological Anatomy

Department of Neurosurgery

National Institute of Oncology of Rabat

**Academic year: 2017-2018**

**Patient Number:**

**Chart Number:**

**1. Demographic characteristics:**

Gender : □ Male □ Female

Age : . . . . . . . . . . . years .

Profession : . . . . . . . . . . . . . . . . . . . . . . . . .

City of origin: . . . . . . . . . . . . . . . . . . . . . . . . . . . .

Adress :

Tel :

**2. Risk Factors:**

□ Alcohol □ Tobacco

□ High blood pressure

□ Diabetes

□ Electromagnetic waves

**3. Family history of cancer: □ Yes □ No □ Type of cancer: ...**

Personal history:

□Medical:... □ Surgical:...

□ Stroke.

-Radiation therapy in childhood □ Yes □ No

-Chemotherapy in childhood □ Yes □ No

**4. Topography of brain glioma:**

□ Temporal □ occipital □ parietal □ deep (thalamus, UCS)

□ Frontal □ FCP □ multiple lobes

**5. Histologic diagnosis:**

□ Astrocytoma grade II

□ Grade III astrocytoma

□ Classic glioblastoma

□ Giant cell glioblastoma

□ Gliosarcoma

□ Epithelioid glioblastoma

□ Oligodendroglioma II

□ Oligodendroglioma III

□ Oligoastrocytoma II

□ Anaplastic oligoastrocytoma

□ Pilocytic astrocytoma

□ Pilomyxoid astrocytoma

□ Subependymal giant cell astrocytoma

□ Xanthoastrocytoma pleomorphic

□ Anaplastic xanthoastrocytoma

**6. Anatomopathological characteristic:**

Mitotic activity: □ Present □ Absent

Necrosis: □ Yes □ No

Endotheliocapillary vascular proliferation: □ Yes □ No

Cell Density: □ Moderate □ High

**7. Immunohistochemical (IHC) profile: 2016 WHO classification:**

- IDH1: □ positive □ negative

- P53: □ positive □ negative

- Ki67: □ high □ low

- NOS □

**8. IHC profile (Research marker):**

- HIF-1- alpha: □ positive □ negative

**9. Clinical and radiological information:**

- Invasive tumor □ Yes □ No

- Karnofsky index

- Imaging (MRI)

- Presence of contrast enhancement □ Yes □ No

**9. Treatment strategy**

| **Surgery** | Yes | Type:  □ Complete □ Incomplete □ Stereotactic biopsy  Date: ……………………………………………….. |
| --- | --- | --- |
|  | No |  |
| **Radiotherapy** | Yes | Total dose: ………………………………………………..  Dose/Fraction : ……………………………………………….  Date : ………………………………………………. |
|  | No |  |
| **Chemotherapy** | Yes | Start date: ……………………………………………….  Type : ………………………………………………..  Protocol : ……………………………………………….. |
|  | No |  |
| **Targeted therapy** | Yes | Start date: ……………………………………………….  Duration: ……………………………………………….  Type : ………………………………………………. |

**10. Follow-up**

| **Clinical examination** | **Cerebral MRI** |  |
| --- | --- | --- |
| **1 mounth** |  |  |
| **3 mounth** |  |  |
| **6 mounth** |  |  |
| **1 year** |  |  |
